# Association between Syphilis Incidence and Dating App Use in Japan

**DOI:** 10.31662/jmaj.2019-0033

**Published:** 2020-03-19

**Authors:** Yosuke Suzuki, Makoto Kosaka, Kana Yamamoto, Tamae Hamaki, Eiji Kusumi, Kenzo Takahashi, Tetsuya Tanimoto

**Affiliations:** 1 Department of Obstetrics and Gynecology, Chiba Medical Center, Teikyo University, Chiba, Japan; 2 Medical Governance Research Institute, Tokyo, Japan; 3 Department of Internal Medicine, Navitas Clinic, Tokyo, Japan; 4 Teikyo University Graduate School of Public Health, Tokyo, Japan

**Keywords:** resurgence of syphilis, sexually transmitted diseases, social media, dating app, Japan

## Abstract

**Introduction:**

The cause of the syphilis resurgence in Japan is still unknown. In this study, we hypothesized that the spread of mobile dating software for use on mobile phones might have contributed to it. We investigated possible contributing factors of the syphilis resurgence in Japan.

**Methods:**

We retrieved the number of reported cases of syphilis, human immunodeficiency virus infection, carbapenem-resistant *Enterobacteriaceae* infection, invasive *Streptococcus pneumoniae* infection, general population, foreign national residents, international overnight guests, detachment-type sex trade shops, physician density, and smartphone penetration rate at the prefectural level in 2017. We also obtained the number of three major dating app users in each prefecture. Using them, we performed association analyses.

**Results:**

The median of syphilis incidence per 100,000 prefectural population was 2.34 (range 0.72 to 12.90). The values of Spearman’s rank correlation coefficients between syphilis incidence and app penetration rates were 0.59 (p < 0.001) for app 1, 0.57 (p < 0.001) for app 2, and 0.56 for app 3 (p < 0.001). The values of correlation coefficient between syphilis incidence and prefectural population (0.50, p < 0.001), foreign national residents per prefectural population (0.46, p = 0.001), and smartphone penetration rate (0.54, p < 0.001) were significant, while international overnight guests per prefectural population (0.19, p = 0.19), sex trade shops (0.24, p = 0.10), and physician density (0.10, p = 0.52) were not. In the multiple regression analyses, the contents of an optimized model included the following two factors: for app 1 penetration rate (0.39, p < 0.001) and the number of sex trade shops per prefectural population (0.18, p = 0.008) with the adjusted R-squared value 0.49 and F value 22.97 (p < 0.001).

**Conclusions:**

Dating app penetration rate was significantly associated with syphilis incidence. The popularization of social media is a possible contributing factor in the syphilis resurgence in Japan. Information concerning the use of social media should be included in future studies on transmission and prevention of sexually transmitted infections.

## Introduction

Among sexually transmitted infections (STIs), the incidence of syphilis has been steadily on the rise worldwide in the past decade, and Japan has been no exception ^(1)^. The Japanese government has mandated that all diagnosed cases of syphilis be reported under the Notifiable Disease Surveillance law since 1948. The annual number of reported syphilis cases throughout the country ranged from 500 to 900 between 2000 and 2012. However, the number has indicated a steady and alarming increase since then: 1228 in 2013, 1661 in 2014, 2690 in 2015, 4575 in 2016, 5826 in 2017, and 7002 (5.6 per 100,000; males, 4588; females, 2414) in 2018 ^(2)^. The rates are getting higher, not only among men but also among young women, particularly in men who have sex with women and women who have sex with men ^(3)^.

Changes of sexual behavior or commercial sex work, insufficient funding for prevention, and poor education during school years are possible causes of the increase in the number of sexually transmitted infections (STIs), but the true reasons for the current syphilis resurgence in Japan still remain unknown ^(1), (3)^. Some hypothesize about its association with the abrupt increase in the number of foreign visitors to Japan, a 3.43 times increase in 2017 compared to 2012. Alternatively, we hypothesized that the explosive spread of specific software applications (apps) on smartphones used to locate and connect with members of the opposite sex (also known as mobile dating apps) launched in 2012, and that had gained wide-ranging popularity since 2013, might have contributed to the significant rise in syphilis cases due to their potential to accelerate casual sex among unfamiliar partners.

In this study, to elucidate the possible contributing factors of syphilis resurgence in Japan, we performed an exploratory statistical data analysis concerning the association between syphilis incidence and dating app penetration rates in 47 prefectures, based on publicly available data.

## Materials and Methods

### Data sources

We retrieved publicly available data concerning the number of new cases of syphilis of any stage at the prefectural level in 2017 as detailed in National Epidemiological Surveillance of Infectious Diseases reports ^(2)^. Similarly, we also retrieved the data on the number of new cases of human immunodeficiency virus (HIV) infection, carbapenem-resistant *Enterobacteriaceae* (CRE) infection, and invasive *Streptococcus pneumoniae* (ISP) infection for reference. In Japan, these diseases are designated as notifiable, and physicians are legally required to report all patients with these diseases.

To obtain an estimate of the number of dating users, we identified three major dating apps available in Japan that reported the number of users in each prefecture. All of the apps were mainly used as a smartphone product and targeted to heterosexual users, men who have sex with women and women who have sex with men.

Between July 18, 2018, and August 2, 2018, three of the authors (MK, TH, and YS) registered with three specific mobile dating apps and retrieved the number of individuals who had accessed the apps within 3 days of research data retrieval in each prefecture to reduce the influence of non-frequent users. Data were unavailable for the access rate per unit time, total application users, and the number of newly registered users, and only the prevalence of application users per population was used for the analyses. In order to estimate the fluctuation in the number of app users, we compared the data retrieved by changing the retrieval date and time. We could not obtain either the number of previous users or the actual number of current users in a unit area smaller than a prefecture.

We also obtained the following data at the prefectural level: the estimate of general population on October 1, 2017, from the Statistics Bureau of the Ministry of Internal Affairs and Communications ^(4)^; the number of foreign national residents in each prefecture in June, 2017, from public data available from the Ministry of Justice ^(5)^; the number of international overnight guests based on the results of the Overnight Travel Statistics Survey for 2017 published by the Japan Tourism Agency ^(6)^; the number of detachment-type sex trade shops for commercial sex workers, which accounted for 94% of registered sex trade shops in 2017, from the statistics of the National Police Agency ^(7)^; the number of physicians who work in medical institutions per prefectural population, from the biennial nationwide survey for physicians, dentists, and pharmacists by the Ministry of Health, Labour and Welfare in 2016 ^(8)^; and the penetration rate of smartphone users, which was derived from the survey of telecommunication services by the Ministry of Internal Affairs and Communications in 2017 ^(9)^.

### Statistical analysis

For a 100,000 population in each prefecture, the incidence of syphilis, HIV infection, CRE infection, ISP infection, app penetration rates, foreign national residents per prefectural population, international overnight guests per prefectural population, physician density, and sex trade shops for commercial sex workers per prefectural population were calculated. Correlation analyses were conducted between the incidence of these infectious diseases and each app penetration rate as well as prefectural population using Spearman’s rank correlation coefficient. Similarly, correlation analyses between the incidence of these infectious diseases and the other explanatory variables were conducted. To exclude the influence of big cities, we also conducted the same analyses by removing 11 prefectures containing cities with 1 million or higher population.

Multiple regression analyses were performed to identify variables to predict the incidence of syphilis. In the regression analyses, the natural logarithms for the syphilis incidence were considered as an objective variable, and the exploratory variables included the prefectural population, the penetration rates of apps, the number of international overnight guests per prefectural population, the number of foreign national residents per prefectural population, the number of sex trade shops per prefectural population, the physician density, and the penetration rates of smartphone users. A stepwise selection method with the Akaike information criterion was performed in order to obtain the best model from a set of predictor variables. The analyses were performed three times independently, using each of the three apps. All the statistical analyses were performed using Microsoft Excel 2016 and the modified version of R (The R Foundation for Statistical Computing, Vienna, Austria) called EZR (Saitama Medical Center, Jichi Medical University, Saitama, Japan) ^(10)^, which allows a graphical user interface. P values < 0.05 were considered as being statistically significant.

An institutional review board approval was not required because we used publicly available and anonymized data only.

## Results

The total number of new cases of syphilis was 5,826 in 2017: 2,111 for stage I, 2,013 for stage II, 117 for the late stage, 1,576 for asymptomatic stage, and 9 for congenital syphilis. Table 1 summarizes the total population, syphilis cases, HIV infection cases, CRE infections cases, ISP infections cases, and the users of each of the three apps used in this study. The fluctuations in the numbers of app users at different time points were less than 5%. Moreover, at the time of data fixation, the number of users were 443,073 (males, 258,299; females, 284,774) for app 1, 178,963 (males, 102,788; females, 76,175) for app 2, and 126,017 (males, 72,741; females, 53,276) for app 3. Table 2 shows median and range of the incidence of syphilis, HIV infections, CRE infections, ISP infections, and the penetration rates of apps 1–3. The median numbers per 100,000 population in each prefecture were 2.34 (range, 0.72–12.90) for syphilis patients, 0.64 (range, 0–3.32) for patients with HIV infection, 1.13 (range, 0.27–3.08) for CRE infection, and 2.42 (range, 0.78–7.28) for ISP infection. The median numbers of users per 100,000 population in each prefecture were 249 (range, 162–744) for app 1, 99 (range, 60–287) for app 2, and 54 (range, 29–243) for app 3. The median numbers per 100,000 prefectural population were 1,155 (range, 383–3,793) for foreign national residents, 18,678 (range, 1,046–166,018) for international overnight guests, 15.1 (range, 5.9–31.3) for the sex trade shops, and 242.4 (range, 160.1–315.9) for physicians at the prefecture level. The median number of smartphone penetration rates was 0.56 (range, 0.46–0.69) at the prefecture level.

**Table 1.** Syphilis and App Users in a Total Number of 47 Prefectures, Japan.

	Total number of 47 prefectures		
	Both sexes	Male	Female
General population (×100,000)	1,267	617	651
Syphilis	5,826	3,931	1,895
HIV infection	1,395	1,319	76
CRE infection	1,660	1,024	636
ISP infection	3,205	1,887	1,318
App 1 users	443,073	258,299	284,774
App 2 users	178,963	102,788	76,175
App 3 users	126,017	72,741	53,276

HIV: human immunodeficiency virus, CRE: carbapenem-resistant *Enterobacteriaceae*, ISP: invasive *Streptococcus pneumoniae*

**Table 2.** Syphilis, App Users, and Other Explanatory Variables at the Prefectural Level, Japan.

	Median (range)		
	Both sexes	Male	Female
Prefectural population (×100,000)	16.3 (5.7–137.2)	7.6 (2.7–67.6)	8.6 (3.0–69.7)
Syphilis incidence	2.34 (0.72–12.90)	3.52 (1.21–18.03)	1.52 (0.19–8.03)
HIV infection incidence	0.64 (0–3.32)	1.24 (0–6.42)	0 (0–0.48)
CRE infection incidence	1.13 (0.27–3.08)	1.32 (0.35–4.25)	0.91 (0.10–3.71)
ISP infection incidence	2.42 (0.78–7.28)	2.93 (0.95–8.32)	1.71 (0–6.27)
App 1 penetration rate	249 (162–744)	230 (151–697)	134 (76–473)
App 2 penetration rate	99 (60–287)	98 (64–338)	59(28–173)
App 3 penetration rate	54 (29–243)	57 (36–209)	38 (20–155)
Foreign national residents per 100,000 population	1,155 (383–3,797)		
International overnight guests per 100,000 population	18,678 (1,046–166,018)		
Detachment-type sex trade shops per 100,000 population	15.1 (5.9–31.3)		
Physician density per 100,000 population	242.4 (160.1–315.9)		
Smartphone penetration rate	0.56 (0.46–0.69)		

HIV: human immunodeficiency virus, CRE: carbapenem-resistant *Enterobacteriaceae*, ISP: invasive *Streptococcus pneumoniae*

**Figure 1.**
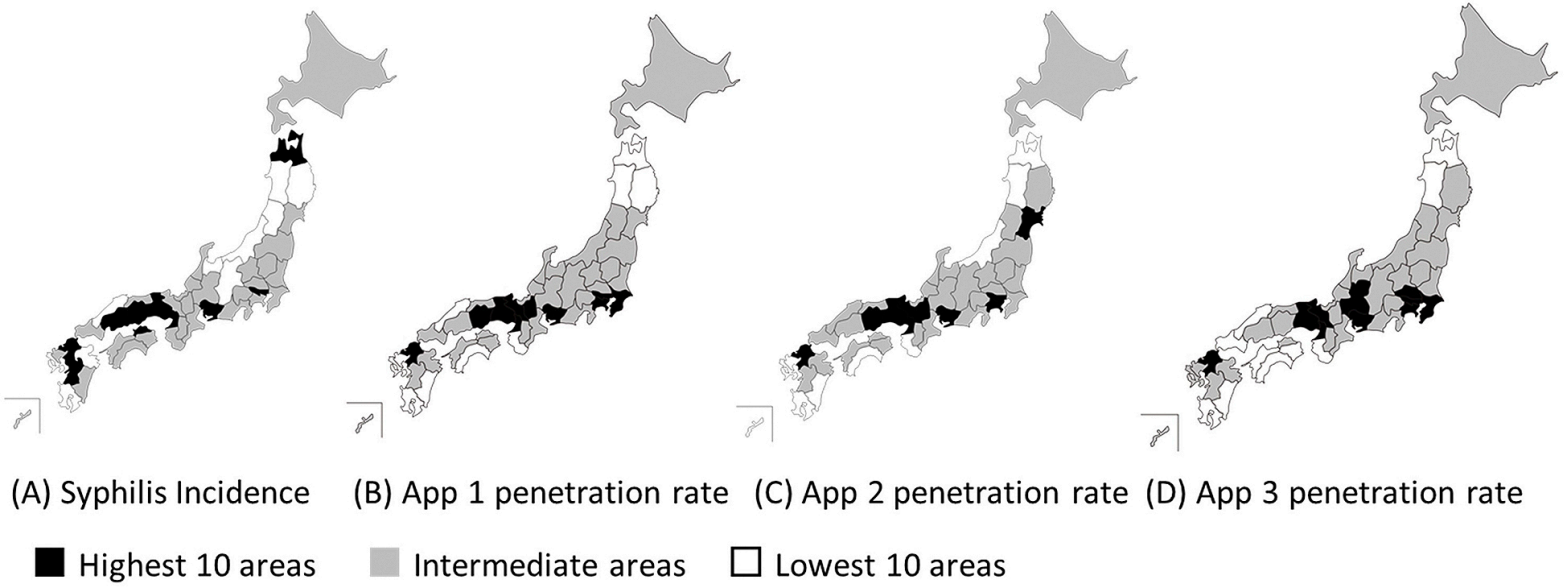
Map A indicates prefectural differences in the annual incidence of syphilis in 2017, while maps B, C, and D show penetration rates of each dating app. Prefectures are classified into three categories: the lowest 10 (white areas), the highest 10 (black areas), and intermediate (gray areas).

**Figure 2.**
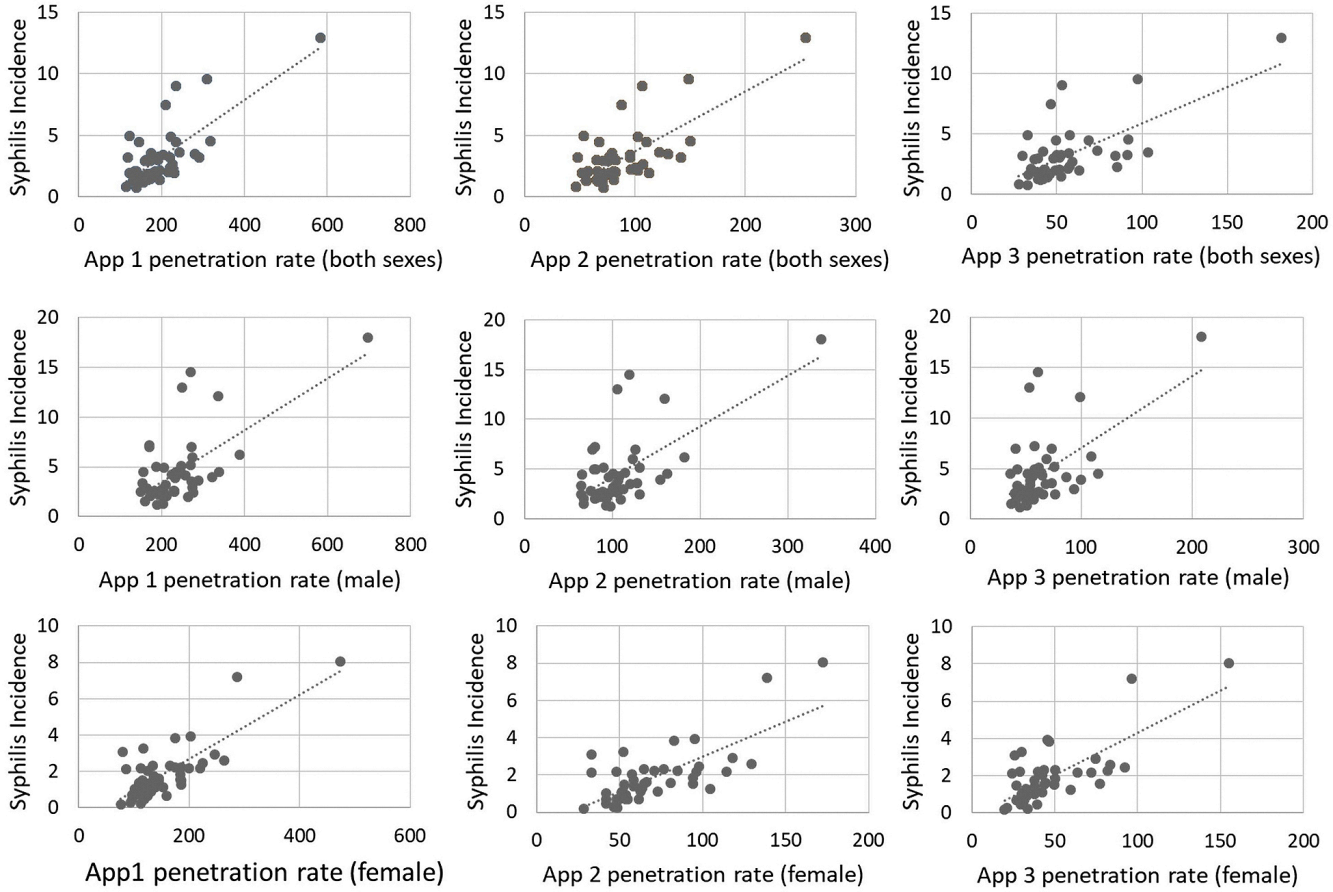
Scatter plots of syphilis incidence and dating app penetration rates.

Figure 1 shows the distribution of the incidence of syphilis and the penetration rates of apps 1 to 3, and these illustrate similar trends, suggesting that prefectures with a high incidence of syphilis (except one prefecture) had high or intermediate penetration rates of apps. Figure 2 shows the scatter plots of the incidence of syphilis and the penetration rates of apps 1–3 and also highlights the associations among them, suggesting that the associations were more probable among females than males.

Table 3 shows the correlation analyses for each item. The values of Spearman’s correlation coefficients between syphilis incidence and app penetration rate were 0.59 (p < 0.001) for app 1, 0.57 (p < 0.001) for app 2, and 0.56 (p < 0.001) for app 3. Among the other six variables, the values of correlation coefficient between syphilis incidence and prefectural population (0.50, p < 0.001), foreign national residents per prefectural population (0.46, p = 0.001), and smartphone penetration rate (0.54, p < 0.001) were significant. On the other hand, the values of correlation coefficient between syphilis incidence and international overnight guests per prefectural population (0.19, p = 0.19), sex trade shops (0.24, p = 0.10), and physician density (0.10, p = 0.52) were not significant. Similarly, the values of correlation coefficient between HIV infection incidence and app penetration rates were significant, while CRE infections and ISP infections had no association with app penetration rates. Analyzing by sex, similar relationships between STIs and app use were found in both male and female, and the relationship was closer in female than in male.

**Table 3.** Spearman’s Rank Correlation Analyses between Syphilis Incidence and App Penetration Rates (47 Prefectures).

(Both sexes)	Syphilis	HIV	CRE	IPS
Population	0.50^*^	0.33^*^	0.02	0.03
App 1 penetration rate	0.59^*^	0.41^*^	0.02	0.29
App 2 penetration rate	0.57^*^	0.34^*^	0.02	0.25
App 3 penetration rate	0.56^*^	0.43^*^	0.02	0.27
Foreign national residents per prefectural population	0.46^*^	0.33^*^	0.01	0.23
International overnight guests per prefectural population	0.19	0.41^*^	0.00	0.16
Detachment-type sex trade shop per prefectural population	0.24	0.38^*^	0.19	0.38^*^
Physician density	0.10	0.27	0.16	0.13
Smartphone penetration rate	0.54^*^	0.48^*^	0.14	0.15
**(Male)**	**Syphilis**	**HIV**	**CRE**	**IPS**
Population	0.44^*^	0.34^*^	0.13	0.01
App 1 penetration rate	0.43^*^	0.30^*^	0.05	0.30^*^
App 2 penetration rate	0.40^*^	0.24	0.06	0.30^*^
App 3 penetration rate	0.43^*^	0.36^*^	0.02	0.26
**(Female)**	**Syphilis**	**HIV**	**CRE**	**ISP**
Population	0.52^*^	0.33^*^	-0.14	-0.02
App 1 penetration rate	0.61^*^	0.44^*^	-0.20	0.11
App 2 penetration rate	0.63^*^	0.41^*^	-0.15	0.03
App 3 penetration rate	0.59^*^	0.47^*^	-0.19	0.12

HIV: human immunodeficiency virus, CRE: carbapenem-resistant *Enterobacteriaceae*, ISP: invasive *Streptococcus pneumoniae*, *P < 0.05

Because it has been reported that in case of syphilis, there are significant differences between the urban and suburban areas ^(11)^, we added correlation analyses removing 11 prefectures containing cities with 1 million or higher population. The remaining area accounts for 44.6% of the national population (including foreign nationals). Table 4 shows that in these analyses for 36 prefectures, the values of correlation coefficients between syphilis incidence and app penetration rate were 0.39 for app 1 (p = 0.039), 0.37 for app 2 (p = 0.051), and 0.37 for app 3 (p = 0.050). Other variables were not significant except for smartphone penetration rate (0.47, p = 0.017). In females, the values of correlation coefficient between syphilis incidence and app penetration rate were 0.40 for app 1 (p = 0.038), 0.45 for app 2 (p = 0.020), and 0.37 for app 3 (p = 0.050). In males, those were 0.22 for app 1 (p = 0.022), 0.13 for app 2 (p = 0.045), and 0.23 for app 3 (p = 0.021).

**Table 4.** Spearman’s Rank Correlation Analyses between Syphilis Incidence and App Penetration Rates (36 Prefectures, Removing Prefectures Containing Cities with 1 Million or Higher Population).

(Both sexes)	Syphilis	HIV	CRE	IPS
Population	0.27	0.17	-0.13	-0.12
App 1 penetration rate	0.39^*^	0.24	-0.21	0.23
App 2 penetration rate	0.37	0.13	-0.22	0.16
App 3 penetration rate	0.37	0.31	-0.25	0.23
Foreign national residents per prefectural population	0.31	0.20	-0.25	0.20
International overnight guests per prefectural population	0.12	0.32	-0.22	0.07
Detachment-type sex trade shop per prefectural population	0.29	0.43^*^	-0.33	-0.46^*^
Physician density	0.01	0.20	-0.01	0.03
Smartphone penetration rate	0.47^*^	0.40^*^	-0.40^*^	0.10
**(Male)**	**Syphilis**	**HIV**	**CRE**	**IPS**
Population	0.25	0.19	0.03	-0.15
App 1 penetration rate	0.22	0.11	-0.11	0.27
App 2 penetration rate	0.13	0.00	-0.13	0.23
App 3 penetration rate	0.23	0.22	-0.16	0.21
**(Female)**	**Syphilis**	**HIV**	**CRE**	**ISP**
Population	0.20	0.16	-0.31	-0.16
App 1 penetration rate	0.40^*^	0.28	-0.45^*^	-0.01
App 2 penetration rate	0.45^*^	0.23	-0.38^*^	-0.13
App 3 penetration rate	0.37	0.36	-0.42^*^	0.05

HIV: human immunodeficiency virus, CRE: carbapenem-resistant *Enterobacteriaceae*, ISP: invasive *Streptococcus pneumoniae*, *P < 0.05

To clarify which factors were able to predict the incidence of syphilis, multiple linear regression analyses were performed using EZR with the Akaike information criterion. Syphilis incidence, population, app penetration rates, foreign national residents per prefectural population, and international overnight guests per prefectural population were log-transformed because their distributions were far from normality. After the stepwise selection method, the results were almost identical regardless of the individual app. When using app 1, as shown in Table 5, the contents of an optimized model included the following statistically significant two factors: the penetration rate for app 1 and the number of sex trade shops per prefectural population. The adjusted R-squared value was 0.49, the F value was 22.97 (P < 0.001), and the standard partial regression coefficient was statistically significant for the penetration rate for app 1 (0.39, p < 0.001) and the number of sex trade shops per prefectural population (0.18, p = 0.008). Similarly, as also shown in Table 5, the optimized models using app 2 and app 3 had almost the same result as app 1.

**Table 5.** The Optimized Models for Syphilis Incidence by Multiple Linear Regression Analyses.

App 1				
	Standard partial regression coefficient	Standard error	T value	P value
Variables				
(Intercept)	0.94	0.063	15.0	0
App 1 penetration rate	0.39	0.064	6.0	< 0.001
Detachment-type sex trade shops per prefectural population	0.18	0.064	2.8	0.008
Adjusted R-squared value = 0.49, F-value = 22.97, P-value < 0.001				
**App 2**				
Variables	Standard partial regression coefficient	Standard error	T value	P value
(Intercept)	0.94	0.065	14.5	0
App 2 penetration rate	0.37	0.066	5.6	< 0.001
Detachment-type sex trade shops per prefectural population	0.19	0.066	2.8	0.007
Adjusted R-squared value = 0.48, F-value = 20.5, P-value < 0.001				
**App 3**				
Variables	Standard partial regression coefficient	Standard error	T value	P Value
(Intercept)	0.94	0.065	14.5	0
App 3 penetration rate	0.37	0.066	5.6	< 0.001
Detachment-type sex trade shops per prefectural population	0.18	0.068	2.6	0.013
Physician density	0.10	0.068	1.4	0.17

Adjusted R-squared value = 0.46, F-value = 13.89, P-value < 0.001

## Discussion

We revealed that in 47 prefectures in Japan, the dating app penetration rate was significantly associated with the increase of syphilis incidence, and the relationship was closer in females than in males. We analyzed three models incorporating three dating apps independently, but this relationship did not change irrespective of the app analyzed. Similarly, apps had significant correlation with HIV infection. For reference, we chose two non-STIs, CRE infections and ISP infections, and observed no association with the app penetration rates except for between ISP infection and male users. The results suggest that the popular use of mobile dating apps might have accelerated both casual sex and promiscuous relationships among unfamiliar couples, resulting in the recent increase in the number of syphilis patients in Japan. On the other hand, the increase in the number of foreign visitors seems to have less significant association with the increase in the number of syphilis patients in Japan, as indicated by the smaller values of correlation coefficient concerning the number of foreign national residents and international overnight guests.

This trend slightly weakened in the analyses that excluded prefectures with large cities. In addition, only app 1 was significant in the model with both sexes, and app 2 was significant in the model with females only. No apps produced a significant result in models with males. Since we surmised that both syphilis incidence and the app penetration rates are higher in urban areas than in the suburbs, we excluded prefectures containing big cities with 1 million or higher population. However, even after the exclusion, the app penetration rates had highest correlation than other factors.

It is unclear why females were more correlated with the incidence of syphilis, although the app penetration rates were lower in females. To evaluate the effect of commercial sex workers, the number of registered shops was used in this study, but there still was little correlation. It may suggest that there are a lot of commercial sex workers using the apps or another way to search for sexual partners without going through the registered shops. Another possibility is the effect of difference in app penetration rate and syphilis prevalence by age and gender. However, it is difficult to verify the correlations with the currently available data.

Previous reports have indicated that in small communities, the incidence of STIs can be affected by the migration of population as well as due to the change in the way couples interact with each other and the use of social media ^(12), (13)^. To the best of our knowledge, this is the first study that has revealed a positive association between the spread of social media and the increase in the incidence of STIs in the whole country.

Sexual behavior can be influenced by various factors, including religion, income, education, and family structure ^(14)^. Although Japan’s population is more than 120 million, it has relatively homogeneous backgrounds among citizens. Additionally, Japan introduced universal health insurance in 1961 and has achieved relatively equal access to quality healthcare in all prefectures ^(15)^. Therefore, the influence of other confounding socioeconomic factors would not be very large in our analysis, which increases the reliability of the findings of our study.

Our study has several limitations. First, we adopted and analyzed the prefecture level data. Although we excluded 11 prefectures containing big cities with 1 million or higher population, relatively large urban and peri-urban areas still exist if the locations were included in this study. Thus, to avoid the selection bias of big cities, it would be best, where possible, to analyze using smaller, municipality level data sets.

Second, our research is an analytical study using publicly available data, and we cannot factor in non-app sexual relationship data. We also cannot specify whether app use resulted in any form of relationship between app users nor what form, sexual or otherwise, any such relationship may have been. Therefore, there may be a different correlation before 2017 and afterward. In future studies focusing on the transmission and prevention of STIs, information on the use of social media is crucial, and in addition to the type and the number of sexual partners, clarification of the behavior patterns of how couples meet would be important in understanding the risk of STIs.

Third, we should be careful about reporting bias. The national surveillance data might have been influenced by increased awareness after the alarming news of the increase in the number of syphilis patients and medical examinations. However, in Japan, the number of syphilis patients had a sixfold increase in 5 years since 2013, and such a significant increase cannot be explained solely by a reporting bias.

Finally, as the number of app users might have included dummy users who market merchandise, there might be a discrepancy between the number of actual users and the number of users included in this study. There is also a possibility that other apps that were not included in this study may show different trends. However, the fluctuations in the number of users at different time points were less than 5%, and all the three apps exhibited similar associations.

In conclusion, we found that the rate of dating app users was closely associated with the increase in the number of patients with syphilis, and the use of social media might be related to the increased risk of STIs due to the increased prevalence of casual sex. Information concerning the use of social media should be included in future studies on transmission and prevention of STIs.

## Article Information

### Conflicts of Interest

KY received personal fees from Nagatanien and Rohto Pharmaceutical, outside the submitted work; EK received personal fees from Daiichi-Sankyo, outside the submitted work; TT received personal fees from MNES Inc., outside the submitted work.

### Acknowledgement

We acknowledge that part of our work has been previously presented at the Annual Meeting of the Japanese Society of Sexually Transmitted Infections and published in the conference abstract (Japanese Journal of Sexually Transmitted Infections, 2018, 29: 253. in Japanese).

### Author Contributions

YS designed this study and wrote the initial draft of this manuscript. MK and TH collected and analyzed the data. KY and EK contributed to the interpretation of the results and the assessment of the validity. KT and TT have critically reviewed the manuscript. All authors approved the final version of the manuscript and agreed to be accountable for all aspects of the work in ensuring that questions related to the accuracy or integrity of any part of the work are appropriately investigated and resolved.

### Approval by Institutional Review Board (IRB)

An institutional review board approval was not required because we used publicly available and anonymized data only.
